# Investigation of Crosslinking Parameters and Characterization of Hyaluronic Acid Dermal Fillers: From Design to Product Performances

**DOI:** 10.3390/gels9090733

**Published:** 2023-09-09

**Authors:** Stefano Pluda, Cecilia Salvagnini, Anna Fontana, Anna Marchetti, Alba Di Lucia, Devis Galesso, Cristian Guarise

**Affiliations:** 1 Fidia Farmaceutici S.p.A., via Ponte della Fabbrica 3/A, Abano Terme (PD), 35031 Padova, Italy; spluda@fidiapharma.it (S.P.); amarchetti@fidiapharma.it (A.M.); adilucia@fidiapharma.it (A.D.L.); dgalesso@fidiapharma.it (D.G.); 2 Department of Biology, University of Padova, 35121 Padova, Italy; ceciliasalvagnini@yahoo.it (C.S.); anna.fontana.10@studenti.unipd.it (A.F.)

**Keywords:** crosslinking, hyaluronic acid, dermal fillers

## Abstract

Despite process similarities, distinctive manufacturing technologies offer hyaluronic acid dermal fillers with different in vitro physicochemical and rheological properties due to peculiar crosslinked hydrogel networks. A better understanding of dermal filler properties could provide specific clinical indications and expectations with more accurate performance correlations. In this study, with an emphasis on the degree of modification, hyaluronic acid concentration and molecular weight, these process parameters were able to modulate dermal filler properties, especially rheology. Moreover, an extensive characterization of commercial hyaluronic acid injectables of the Hyal System line was described to present product properties and help to elucidate related clinical effects. Standardized methodologies were applied to correlate in vitro parameters with feasible clinical indications. In view of an optimized dermal filler design, the results of the extrudability measurements allowed the quantification of the effect of hydrogel composition, rheological properties and needle size on injectability. Composition, dynamic viscosity and needle size showed an impactful influence on hydrogel extrudability. Finally, the positive influence of 200 KDa hyaluronic acid in comparison to fragments of ether-crosslinked hyaluronic acid on fibroblast recognition were shown with a migration assay.

## 1. Introduction

Hyaluronic acid (HA) is a linear heteropolysaccharide composed of D-glucuronic acid and N-acetyl-D-glucosamine units. It is a member of the glycosaminoglycan family and an essential component of the extracellular matrix. Because of its unique biocompatibility, biodegradability and hydrophilicity, HA is widely used as a component of dermal fillers [1,2]. HA dermal fillers were at the second place of non-surgical procedures worldwide in 2021 after botulinum toxin injections, with an increase of 30% from the previous year and a total of 5.3 million treatments [3]. A common strategy to drastically enhance the in vivo HA persistence is to link the polymer chains together to create a crosslinked hydrogel with improved resistance to enzymatic and radical degradation [4]. Chemical crosslinking allows for obtaining a hydrogel with a constrained three-dimensional network from a viscous solution. Crosslinking density is correlated with hydrogel strength, which is the result of covalent crosslinking and intermolecular physical interactions, such as hydrogen bonding and physical entanglements [4,5]. In vitro hydrogel characterization, with a specific focus on viscoelastic behaviors, has been extensively reported, in order to compare hydrogel properties, correlate them with clinical performances and assist clinicians in selecting the appropriate attributes [5,6,7,8,9]. However, despite principal manufacturers of dermal fillers using the same crosslinking agent, 1,4-butanediol diglycidyl ether (BDDE), which is the most commonly used reagent for HA crosslinking in alkaline conditions, different technologies afford a plethora of dermal fillers with peculiar characteristics [7,10]. Parameters of the manufacturing process, such as crosslinking degree, crosslinking conditions, molecular weight of the starting HA and fragmentation, severely impact filler characteristics [5,11]. In this study, principal parameters of the manufacturing process that impact filler characteristics, and particularly rheological properties, were evaluated. Moreover, the characterization of commercial dermal injectables of the Hyal System line was assessed through standardized methodologies to describe their rheologic and physicochemical properties. Finally, extrudability was investigated and correlated with hydrogel rheology and needle characteristics. Also, effects of linear and partially depolymerized crosslinked HA were evaluated in a cell migration assay to investigate the effect of different polymer structures.

## 2. Results and Discussion

### 2.1. Crosslinking Parameters Modulate Hydrogel Rheology

Variations in manufacturing processes and post-crosslinking modifications affect dermal filler characteristics, and particularly rheological properties [12]. Some technologies for the production of HA dermal fillers that are currently available on the market and related product-by-process claims are summarized in Table 1 in order to highlight some relevant process variables.

In order to evaluate the impact of important process variables on the viscoelastic behavior of hydrogels, HA solutions were crosslinked at diverse theoretical crosslinking degrees (5–20% mol/mol), polymer concentrations (65–300 mg/mL) and molecular weights of HA starting material (200, 700 and 1800 KDa).

**Figure 1:**
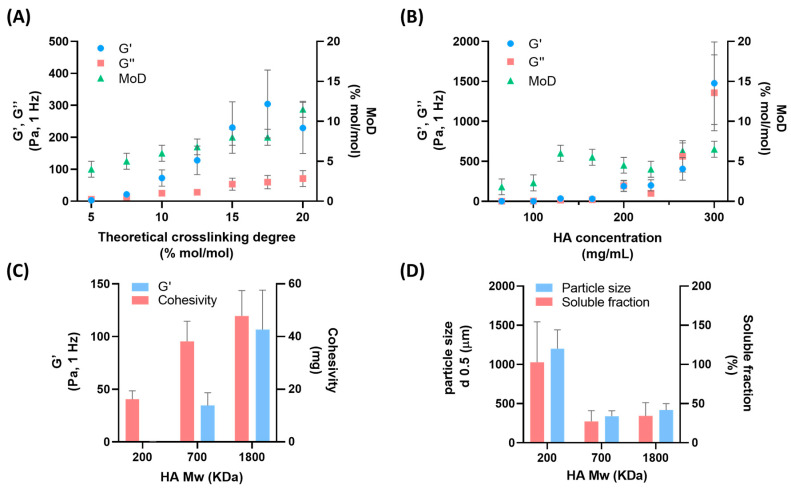
Elastic modulus (light blue dots), viscous modulus (red squares) and degree of modification (green triangles) of HA hydrogels with theoretical crosslinking degree from 5 to 20% mol/mol vs. HA repeat unit (**A**) and HA reaction concentration from 65 to 300 mg/mL (**B**). Elastic modulus and cohesivity (**C**), and particle size and soluble fraction (**D**) of HA hydrogels with starting molecular weights (200, 700 and 1800 KDa). Mean ± DS; n = 2.

The rheological characterization, reported in Figure 1A, of hydrogels obtained with crosslinker amounts from 5 to 20% mol/mol vs. the HA repeat unit of the polymer evidenced the correlation of elastic and viscous moduli with the degree of modification. In accordance with our previously reported data, a linear correlation between theoretical crosslinking degree and degree of modifications was observed [15]. Moreover, chemical crosslinking via covalent bonding increased hydrogel strength with a prevalence of the elastic contribution [5]. The theoretical crosslinking degree allowed the modulation of the viscoelastic properties of HA hydrogels, in accordance with our previously published data [16,17]. As a second point, polymer concentrations during the chemical reaction were explored from 65 to 300 mg/mL of HA in alkaline conditions, and hydrogels after the neutralization step were finally hydrated to the same concentration, equal to 25 mg/mL. It is known that polymer concentration and crosslinking density affect rheological properties and clinical performances of dermal fillers [5,18]. However, to the best of our knowledge, no studies on the effects of HA concentration during the reaction have been previously reported. We hypothesized that HA concentration during the crosslinking reaction may impact crosslinking efficiency, crosslinking density and hydrolysis kinetics due to the alkaline condition. We observed a severe impact on the rheology of hydrogels, reported in Figure 1B, not correlated with the degree of modification (MoD). Low values of elastic and viscous moduli were obtained with concentrations of HA in reaction lower than 130 mg/mL. With a percentage elasticity inferior to 10% and quantitative soluble fractions, viscous solutions with low mechanical strength rather than hydrogels were obtained (Figure S1A). These reaction conditions did not allow the efficient crosslinking of polymer chains, as supported by the low values of MoD. On the other hand, at higher reaction concentrations of HA, the MoD became constant but with a drastic increase in rheology. Likely, higher viscoelastic values may be explained by a different crosslinking density or an entangled network. This explanation is supported by a reduction in the soluble fraction of HA (non-crosslinked HA) as the reaction concentration increases, as reported in Figure S1A.

Another important parameter for the modulation of the viscoelastic behavior of hydrogel dermal fillers is the starting molecular weight of HA. Crosslinked hydrogels obtained from HA with longer chains, at a comparable concentration and crosslinking degree, afford more constrained three-dimensional networks because of the greater entanglement [18,19]. Xue and coworkers reported the impact of a low and high molecular weight of HA hydrogels on the mechanical properties and in vitro degradation [18]. Moreover, Vycross^®^ and Hylacross^®^ technologies (Allergan Aesthetics, AbbVie company) claim to use different proportions of a low and high Mw of HA to achieve products with different properties [18]. HA with an Mw of 200, 700 and 1800 KDa were crosslinked by applying the same reaction conditions, leading to hydrogels with a distinct elasticity and cohesivity, as reported in Figure 1C. Hydrogels showed comparable degrees of modification (Figure S1B); however, elastic modulus and cohesivity values increased with Mw. The length of the polymer tuned the ability of the hydrogel to store energy during deformation along with the internal adhesion forces holding together the hydrogel particles. Moreover, hydrogels obtained from low-Mw afforded particles with larger size and greater solubility, compared to those obtained from high-Mw HA, likely as a result of a less constrained network (Figure 1D). As previously reported by La Gatta and coworkers, chemical bonds between polymer chains of crosslinked hydrogels are influenced by HA concentration and Mw, affecting swelling characteristics [20].

### 2.2. Characterization of Hyal System Dermal Products

In this work, the properties of Hyal System dermal products were investigated in vitro in order to offer an exhaustive overview, to correlate them with clinical performances and to guide clinicians [12]. As reported in the leaflets, Hyal System 1.8% is a linear HA hydrogel based on Molecular Sieving Technology^®^ that is indicated for skin hydration and revitalization with a medium-term corrective effect due to its defined residence time. Hyal System ACP (Auto-Crosslinked Polymer) is a crosslinked hydrogel without the presence of chemical bridges, based on Auto-Crosslinked Polymer technology^®^ [16]. Crosslinking is based on the internal esterification reaction and increases the HA residence time after injection [4]. The product is indicated to improve skin turgidity and elasticity, with limited volumetric indications [21]. Hyal System DUO is composed of two types of crosslinked HA, with ester and ether crosslinking, for long-term correction and the filling of medium to deep skin defects, based on Multi-Crosslinked Hyaluronan Technology^®^. Finally, Hyal Systems LIP and VOL are volumetric gels with ether crosslinking, based on Multi-Crosslinked Layering Hyaluronic Acid technology^®^. Hyal System LIP is indicated for the correction of medium and deep facial skin sagging and lip remodeling. Hyal System VOL is indicated for volume restoration and correction of deep face skin sagging. Products of Hyal System Line lie in the Weight—Concentration—Technology (WCT) Concept™, that is the combination of these three variables in order to reach targeted key features. Linear HA and two types of crosslinked HA hydrogels offer different performances in biological systems [22]. Briefly, native HA is employed to help the skin regain elasticity, turgor and moisture [23], and crosslinked HA is employed to restore volume loss, fill fine wrinkles with increased residence time.

#### 2.2.1. Rheological and Physicochemical Characterization

Hydrogel products were characterized using standardized methodologies. Rheological and physicochemical properties are reported in Table 2.

**Figure 2:**
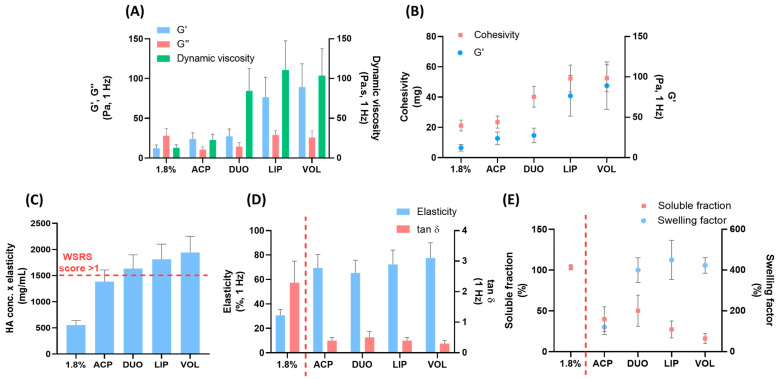
Properties of Hyal System dermal fillers (1.8%, ACP, DUO, LIP, VOL): Elastic modulus, viscous modulus and dynamic viscosity (**A**). Cohesivity and elastic modulus (**B**), HA conc. x elasticity (values above the dashed line: WSRS score > 1) (**C**). elasticity and tanδ, (**D**), and soluble fraction and swelling factor (**E**) (values to the left of the dashed line: linear HA, to the right: cross-linked HAs). Mean ± DS; n = 3.

In Figure 2A, rheological data of viscoelastic modulus and dynamic viscosity of the hydrogels are reported. Importantly, the behavioral assessment of fillers in response to mechanical stress at low frequencies provides clinically relevant indications [18,23]. Hydrogel products showed different elastic properties, or in other words, disparate abilities to store energy during deformation. On the contrary, comparable viscous moduli were observed despite the different typologies or absence of crosslinking. Finally, increasing dynamic viscosity values translated into a resistance to flow and correlated with product extrudability and ease-of-injection [18]. In Figure 2B, cohesivity values and elastic moduli are reported for the five hydrogels, showing an increasing trend, and more elastic hydrogels afforded higher cohesivity. Elasticity is related to the number of covalent bonds between HA chains, and cohesivity is caused by noncovalent interactions [18]. This trend contrasts with the inverse relationship between gel strength and cohesion, previously reported by Edsman 2015 [24] and coworkers. However, cohesion is profoundly affected by hydrogel concentration [25] and this trend may be affected by differences in the composition and concentration of Hyal System hydrogels.

Clinical persistence data do not translate with rheological properties of the various fillers [26]. On the other hand, Falcone and coworkers reported a linear correlation between the clinical score of the 6-month improvement in the wrinkle severity rating scale (WSRS) and the product of HA concentration and elasticity [27]. In Figure 2C, the product of HA concentration and elasticity of the five hydrogels is reported. Considering a WSRS score superior to 1 for values of 1500 mg/mL, it is possible to observe that three products (DUO, LIP, VOL) afforded superior values. In other words, products containing hydrogels crosslinked via ether bonds are designed to assess the long-term effectiveness of soft-tissue augmentation and other facial contouring procedures. This projection is correlated with the distinct product uses and indications. Finally, the differences between linear and crosslinked hydrogels are highlighted by the plots reported in Figure 2D,E. In Figure 2D, elasticity and tan delta values of five products are reported and the rheological parameters highlight their inverse correlation. Hydrogels with linear HA have a tan delta greater than 1 and lower elasticity percentage compared to the crosslinked counterparts [18]. Crosslinked fillers afford a tan delta value lower than 1 because the elastic behavior under low shear stress is dominant over the viscous behavior [12,18]. Moreover, crosslinking affects the water uptake of hydrophilic polymers [18]. As reported in Figure 2E, hydrogel 1.8% composed of linear HA is completely soluble and not able to swell. On the contrary, crosslinked hydrogels showed an inverse trend between soluble fraction and swelling behavior. The swelling factor is useful to predict the initial volumization of an implanted gel and is related to the degree of crosslinking and HA concentration [18]. All these data are useful to define the range of rheological properties, cohesivity, particle size, HA soluble fraction, hydration capacity and degree of modification that characterize these products. Different properties translate to different in vitro and clinical behaviors, highlighting the differences among marketed products.

**Figure 3:**
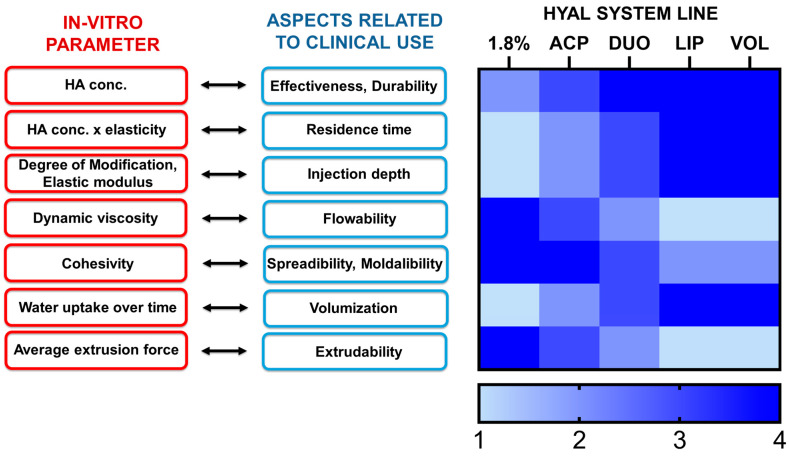
Schematic representation of the correlation between in vitro parameters and clinical aspects of Hyal System dermal fillers (1.8%, ACP, DUO, LIP, VOL).

Correlations between in vitro parameters and features of HA-based dermal products are outlined in Figure 3 [8,12,27,28]. In order to align characteristics to optimal clinical performance, the data of parameters of Hyal System dermal fillers reported in Table 2 and Table 3 were evaluated on a scale from 1 to 4, according to the heat map reported in Figure 3. Nonetheless, for the safe use of fillers, it is critical to provide informed product specifications and applications to trained physicians [29].

#### 2.2.2. Extrudability

The quantification of the extrusion force of HA fillers through a needle, without taking into account tissue resistance, is crucial for performance evaluation and has a clinical importance for physicians [7,28]. In a typical extrusion force curve, force is plotted as a function of the displacement of the syringe. Slope is calculated until the extrusion force increases or the flow begins. Then, at the plateau, the viscous regime dominates, and extrusion force is calculated as the average value [7,28]. Nonetheless, the extrusion of the product through a needle is allowed through hydrogel sizing or fragmentation down the gel mass, which are performed by passing the gel through a series of sieves and screens [11,12]. The results of the average extrusion force and extrusion force slope of Hyal System products are reported in Table 3. Each product was tested using the needles provided into the packaging.

**Figure 4:**
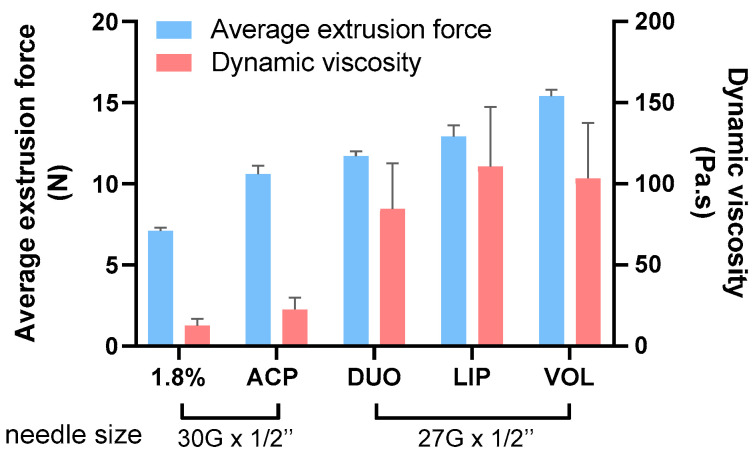
Average extrusion force and dynamic viscosity of Hyal System dermal fillers (1.8%, ACP, DUO, LIP, VOL) with needle reference. Mean ± DS; n = 3.

The reported values allowed the characterization of the extrudability range of the products. Comparable values of average extrusion force were found with previous published data of skin quality boosters and fillers extruded through 30 G-inch needles [30,31,32]. However, superior extrudability was observed for VOL and LIP when compared to data reported by La Gatta et al. extruded through 27 G-inch needles [33]. These data relate to the different product characteristics and provide useful values for clinical indications. An ideal HA filler is one with a low extrusion force, allowing for easy and precise dosing during injection [18]. Moreover, the force required for extrusion is related to the viscosity of the product, which is related to the concentration, degree of crosslinking, amount of crosslinked and non-crosslinked HA, sizing method, particle size, size distribution and crosslinking parameters [28]. Hydrogel viscosity affects extrudability because a filler is subjected to high levels of shear stress during injection. The average extrusion force of five Hyal System dermal products was determined at a constant syringe geometry and extrusion rate and plotted with dynamic viscosity values, as reported in Figure 4.

In conclusion, dynamic viscosity is a useful parameter to evaluate the dependence of product flowability on hydrogel characteristics; however, the extrusion force allows extrudability to be evaluated while also taking syringe geometry, extrusion rate and needle size into account [18,28].

### 2.3. Linear HA and ACP Improve Extrudability

A reported manufacturing strategy to modulate the rheology of hydrogel and increase the extrudability of dermal filler is the introduction of linear HA [28,34], but no data on molecular weight and concentration are reported.

**Figure 5:**
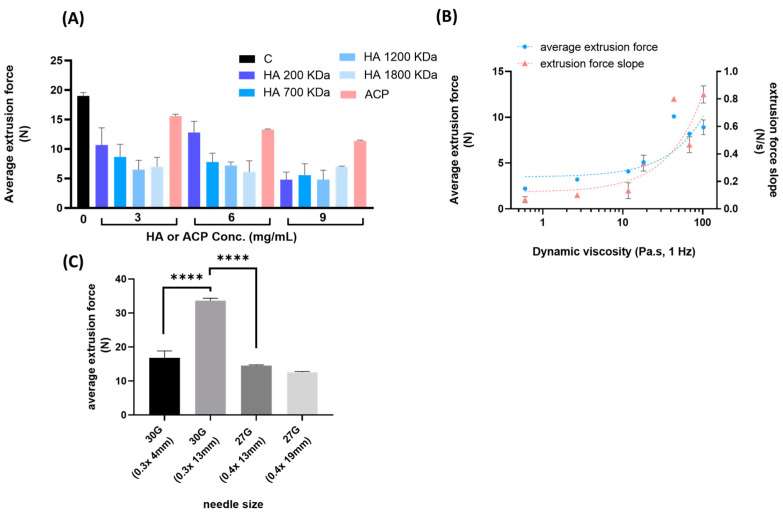
Extrusion values of ether-crosslinked hydrogels with linear HA (200, 700, 1200, 1800 KDa) or ACP at different concentrations (**A**). Extrusion values of ether-crosslinked hydrogels with different dynamic viscosity values (0.6–102.3 P·s at 1 Hz) (**B**) and needle sizes (30 G and 27 G) (**C**). Mean ± DS; n = 3; **** *p* < 0.0001.

Here, we investigated the effect of linear HA and ACP on the extrudability of ether-crosslinked hydrogel through a 30 G needle (0.3 × 4 mm). Linear HA with a molecular weight in the 200–1800 KDa range, and ACP were tested at different concentrations with a total HA concentration of 25 mg/mL. As reported in Figure 5A, the addition of HA effectively reduced the extrusion force with the dependence of Mw and concentration. This result may be explained by the hypothesis that longer chains and higher concentrations of linear HA decrease the number of interactions between crosslinked chains of the polymer [35]. Also, ACP improved the extrudability of the hydrogel in a concentration-dependent manner. The addition of a crosslinked hydrogel is less effective compared to linear HA, but it also contributes to modulate the rheology of the hydrogel.

### 2.4. Dynamic Viscosity and Needle Size Influence Hydrogel Extrusion

Rheological characterization of the dermal filler allows for evaluation of the response of the material to mechanical forces during the injection and once implanted [6,18]. Characteristic shear thinning behavior allows the gels to flow easily during extrusion and remain in place after implantation [36]. To the best of our knowledge, there are no data reported on the extrusion force profile that correlates to dynamic viscosity and needle size [7]. Hydrogels with different values of dynamic viscosity (0.6–102 Pa·s at 1 Hz) were tested to determine average extrusion values and extrusion force slopes using a 27 G needle, as reported in Figure 5B. Hydrogels were obtained with the variables of the manufacturing process discussed in Section 2.1 in order to include various properties. As expected, extrusion and slope values were in accordance and proportionally correlated with dynamic viscosity. These data confirmed that hydrogels with a low resistance to flow afforded a better ease-of-injection. Finally, the impact of needle size on HA hydrogel extrudability was tested. As expected and reported in Figure 5C, we observed a significant and comparable decrease in extrusion force with decreasing length (13 and 4 mm) for the 30 G needle and increasing width (0.3 and 0.4 mm) for the 30 G to 27 G needle. However, no difference was observed for extrusions with the 27 G needle at different lengths (13 and 19 mm).

### 2.5. Cell Migration with Linear and Crosslinked HA

**Figure 6:**
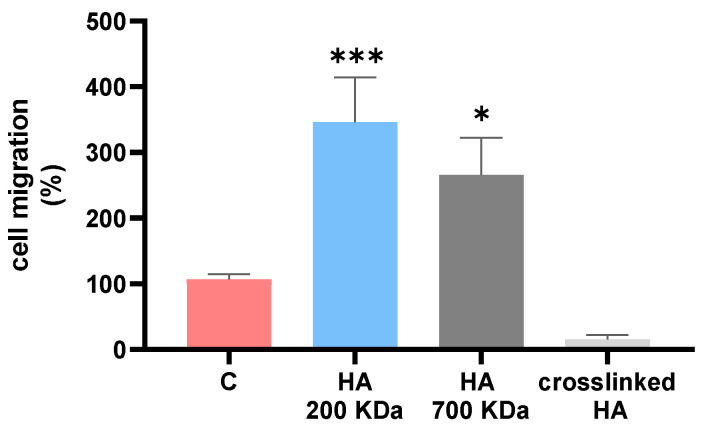
Murine fibroblast migration plot after treatments with HA 200 KDa, 700 KDa and depolymerized BDDE-crosslinked HA after 6 h. Mean ± DS; n = 3; * *p* < 0.05 and *** *p* < 0.001.

As reviewed by Maytin, HA and dermal fillers are able to regulate the differentiation of skin cells and ECM remodeling through the interaction of extracellular HA with CD44, a major cell-surface receptor [37]. BDDE-crosslinked HA hydrogels offer a superior chemical stability compared to crosslinked ACP hydrogels under degradative conditions. ACP hydrogel is obtained from the esterification of 200 kDa HA and designed with cleavable ester bonds. On the other hand, BDDE-crosslinked HA hydrogels are susceptible to hyaluronidase and ROS degradation with the subsequent release of partially crosslinked fragments [16,38]. For this reason, linear HA with molecular weights of 200 and 700 KDa, as well as depolymerized BDDE-crosslinked HA, were tested in a migration assay. We observed that the chain length and crosslinking influence fibroblast migration abilities, likely affecting substrate availability and cell recognition, as reported in Figure 6. Crosslinked HA hinders cell migration, whereas both HA fractions (particularly HA 200 kDa) facilitate migration, resulting in a significantly increased gap closure after 6 h compared to the control. With regard to a previous study reporting a proliferative effect of ACP on keratinocytes [22], this may evidence the importance of the molecular weight of the released HA from hydrogel degradation. Further studies are necessary to investigate the effect of hydrogels on collagen synthesis and ECM remodeling.

## 3. Conclusions

A series of crosslinked hydrogels were prepared and characterized in order to investigate the parameters of the crosslinking process that impact the product rheology of HA dermal fillers. Degree of crosslinking, polymer concentration and molecular weight were able to modulate the physicochemical properties and influence hydrogel networks via covalent crosslinking and physical interactions. Moreover, an extensive characterization of commercial dermal products of the Hyal System line was provided to describe product characteristics. Injectability was investigated via extrudability measurements and correlated with dynamic viscosity. Also, hydrogel extrudability was evaluated in relation to composition, dynamic viscosity and needle size. The results showed that linear HA, at different chain lengths, and ACP improve extrudability in a concentration-dependent manner. This study provides relevant information on injectability for optimal dermal filler design. Finally, HA chain length and crosslinking were able to influence migration abilities of fibroblasts, likely affecting substrate availability and cell recognition. In conclusion, the investigation of the crosslinking parameters, together with the characterization of the rheological, physicochemical and extrudability properties of the Hyal System products, has been explored and discussed in order to provide clinicians with details on the product design.

## 4. Materials and Methods

### 4.1. Materials

Hyaluronic acid sodium salt (HA), auto-crosslinked polymer (ACP) sodium salt and Hyal System dermal fillers (1.8%, ACP, DUO, LIP, VOL) were supplied by Fidia Farmaceutici S.p.A (Abano Terme, Italy). All other reagents were purchased from Merck KGaA (Darmstadt, Germany) and no further purifications were made. Highly purified water was obtained through deionization and filtration with a Millipore purification apparatus.

### 4.2. Preparation of Crosslinked HA Hydrogels

HA-crosslinked hydrogels were prepared according to the protocol previously reported [17] and modified as described later. HA sodium salt (2.0 g, 700 kDa) was dissolved in NaOH (15.28 mL, 0.25 M, 0.77 eq vs. HA repeat unit), reaching a concentration of 130 mg/mL. Subsequently, BDDE (0.101 g, 0.1 eq vs. HA repeat unit) was added for a theoretical crosslinking degree of 10% mol/mol. The solution was left at room temperature for 1 h and stirred occasionally. The reaction was carried out in a thermostatic bath for 4 h at 45 °C, and then neutralized with HCl (38.2 mL, 0.1 M) and PBS (8 mL, 116 mM, NaCl 911 mM, pH 6.5) and 18.5 mL of water, reaching a final polymer concentration of 25 mg/mL. The hydrogels were allowed to swell overnight at 20 °C. Then, they were first roughly filtered with a 1000 μm filter (Swinnex^®^, Merck, Darmstadt, Germany) and, with a stainless-steel mesh (100 μm), divided into glass syringes (1 mL of Hypak SCF™ PRTC, Becton Dickinson, Milano, Italy) and steam-sterilized.

Syntheses with theoretical degrees of crosslinking of 5, 7.5, 12.5, 15, 17.5 and 20% mol/mol were performed by adding 50, 76, 126, 151, 177 and 202 mg of BDDE, respectively, and following the synthetic procedure previously reported. The % mol/mol is calculated as mol of BDDE/mol of HA repeat unit × 100. Syntheses with HA reaction concentrations of 65, 100, 130, 165, 200, 230, 265 and 300 mg/mL were performed using 1.0, 1.5, 1.9, 2.4, 2.9, 3.4, 3.9 and 4.4 g of HA, respectively, with a theoretical crosslinking degree of 10% mol/mol and following the synthetic procedure previously reported. Syntheses with HA molecular weights of 200 kDa and 1900 kDa were performed with a theoretical crosslinking degree of 10% mol/mol and following the synthetic procedure previously reported.

### 4.3. Rheological Measurements

Approximately 1 mL of hydrogel was analyzed using a rheometer (MCR 92, Anton Paar, Rivoli, Italy) equipped with a parallel plate measuring system (PP50, Anton Paar) and a fixed, smooth plate (Anton Paar, Ø = 50 mm, I-PP50/SS) at 25 °C with a measuring gap of 0.102 mm, as previously reported [17]. The G’ (elastic modulus) and G” (viscous modulus) were measured (in Pa) from 0.07 to 30.0 rad/s at a fixed strain value of 10% (an initial sweep strain with an oscillatory shear strain of increasing amplitude, at a constant frequency of ω = 1 Hz, was applied to determine the region of linear response of the sample: at 10% the viscoelastic range is linear). The percentage of elasticity was calculated as described by Santoro et al. [39].

### 4.4. Determination of Degree of Modification

The degree of modification was measured on a Bruker 400 MHz Advance III HD NMR spectrometer (Billerica, MA, USA) equipped with a 5 mm BBIz-grade probe and calculated as previously described [15,40].

### 4.5. Cohesivity

Cohesivity of hydrogel samples was evaluated via drop weight assay, as described by Edsman et al. [24]. The syringe with the hydrogels was centrifuged at 2500 rpm for 5 min to remove the bubbles. The syringe was equipped with an 18 G needle with a plane orifice. The gel was extruded with a constant flow rate (200 mg/min) with a Syringe pump (Harvard apparatus, Holliston, MA, USA). The first drops of hydrogel were discarded until a constant flow was obtained, and then 10 drops were collected and weighed to calculate the average weight of a single drop.

### 4.6. Particle Size

The particle size of the samples was measured, as reported by La Gatta et al. [33]. The hydrogel sample was dispersed in water and the particle size distribution was measured using a Mastersizer 2000 (Malvern Instruments, Malvern, UK). The particle size value was extrapolated as d(0.5).

### 4.7. Soluble fraction

The soluble fraction of the hydrogels was quantified as reported by La Gatta et al. [41]. Sample was diluted in PBS (116 mM, NaCl 911 mM, pH 6.5) to obtain a concentration of 0.80 mg/mL and left under stirring overnight. After centrifugation (2500 rpm for 5 min), the supernatant was collected and filtered (0.20 μm nylon filter) and HA concentration was determined via Viscotek TDA (Milano, Italy) analysis as previously reported [16].

### 4.8. Swelling Factor

The swelling factor was measured by applying minor variations to the method reported by La Gatta et al. [41]. About 0.1 g of each hydrogel was weighed and then 900 μL of PBS (116 mM, NaCl 911 mM, pH 6.5) was added. The samples were left under rotation overnight to reach the swelling equilibrium and then centrifuged at 3000 rpm for 5 min. The supernatant was stained for visualization with toluidine blue (10 μL, 0.1% *w*/*w*) and removed. The swollen hydrogel was weighed, and the swelling factor was calculated as initial gel weight/swollen gel weight × 100.

### 4.9. Extrusion Force Measurements

Extrusion force was measured using a texture analyzer (TX-700, Lamy rheology, Lyon, France) with a support suitable for the placement of the syringe. Fillers were extruded at a fixed compression rate of 10 mm/min. Extrusion force slope was determined until the increase in the signal, and average extrusion force was determined as average value of signal at plateau.

The extrudability of ether-crosslinked hydrogels with linear HA or ACP was evaluated at a concentration of 25 mg/mL of total HA. Ether-crosslinked hydrogel was obtained according to the procedure previously reported and used as a control. HA solutions at molecular weights of 200, 700, 1200 and 1800 KDa, and ACP hydrogels were mixed with ether-crosslinked hydrogels with a final concentration of 3, 6 and 9 mg/mL (22, 19 and 16 mg/mL of HA-BDDE-crosslinked hydrogel, respectively). Hydrogels were allowed to rest overnight at 4 °C before extrusion at room temperature through a 30 G needle. Extrusion force measurements of hydrogels with dynamic viscosity values of 0.6, 2.7, 11.7, 18.2, 43.6, 68.1 and 102.3 Pa·s at 1 Hz were tested using a 27 G (0.4 × 13 mm) needle. Extrusion force measurements of hydrogels with elastic and viscous moduli of 143.8 Pa and 48.1 Pa, respectively, and dynamic viscosity of 108 Pa·s at 1 Hz were determined using 27 G and 30 G needles.

### 4.10. Cell Culture

The murine fibroblasts BALB/3T3 clone A31(ATCC) were cultured in Dulbecco’s Modified Eagle’s Medium (DMEM, Gibco, Waltham, Massachusetts) supplemented with 10% fetal bovine serum (Gibco) at 37 °C in a humified 5% CO_2_ atmosphere. Culture medium was replaced every other day and cells were subcultured when they reached 80% of confluence.

### 4.11. Migration Assay

Migration assay was performed using silicone inserts with defined cell-free gap (Culture-Inserts 2 Well for self-insert, Ibidi, Gräfelfing, Germany) according to the manufacturer’s instructions. Cells were allowed to migrate into the gap once the insert was removed. The day before the experiment, fibroblasts (70 µL, 3 × 10^5^ cells/mL) were seeded in the two compartments of the insert and left to incubate at 37 °C, 5% CO_2_. After 24 h, inserts were removed and, after a wash in Dulbecco’s phosphate-buffered saline 1X (EuroClone, Milano, Italy), medium was replaced with basal medium (DMEM 0.5% FBS) as a control or with basal medium conditioned with HA 200 KDa (10 mg/mL), HA 700 KDa (10 mg/mL) or BDDE-crosslinked HA (1 mg/mL) treated at 105 °C for 8 h. A microscopical observation of the gap was performed at the beginning and 6 h after the insert removal using a fluorescence microscope (Leica DMi8, Wetzlar, Germany). The distance between each edge of the septum was measured using the software ImageJ 1.52a, and the measure of the gap closure and of the cell migration was calculated as [(gap closure on control condition (0.5% FBS)—gap closure on test condition)/gap closure on control condition (0.5% FBS)] × 100.

### 4.12. Statistical Analysis

Measurements were performed at least in duplicate. All data are presented as mean ± standard deviation. Statistically significant differences were evaluated through a one-way ANOVA with Tukey’s multiple comparison test using GraphPad Prism 9 and considering *p*-value significance of * *p* < 0.05, ** *p* < 0.01, *** *p* < 0.001 and **** *p* < 0.0001.

## Figures and Tables

**Table 1:** A representative list of manufacturing technologies and related process variables used for HA dermal filler production.

Technology	Manufacturer	Product-by-Process Claims	Patent Reference	Literature Reference
Non-Animal Stabilized HA (NASHA^®^)	Galderma	- sizing	EP3013866B1	[5]
Optimal Balance Technology (OBT^®^)		- degree of crosslinking - sizing	WO2018104426A1	[11]
Hylacross^®^	Allergan	- high proportion of high to low HA molecular mass	US 8357795 B2	[13]
Vycross^®^		- high proportion of low to high HA molecular mass	US 7741476 B2	[14]
Cohesive Polydensified Matrix (CPM^®^)	Merz	- double HA addition	EP1968711B1	[9]
Resilient HA (RHA^®^)	Teoxane	- degree of crosslinking - HA concentration - reaction in pouch	EP2429486B1	[10]

**Table 2:** Rheological and physicochemical properties of Hyal System dermal products (1.8%, ACP, DUO, LIP, VOL).

Parameter	Unit	Hyal System				
		1.8%	ACP	DUO	LIP	VOL
Concentration	mg/mL	18	20	25	25	25
	% linear HA	100	0	0	7 ± 3	4 ± 3
Composition	% ester crosslinked HA	0	100	25 ± 5	0	0
	% ether crosslinked HA	0	0	75 ± 5	93 ± 3	96 ± 3
G′ (1 Hz)	Pa	12.3 ± 4.1	23.9 ± 7.9	27.5 ± 9.1	76.5 ± 25.2	89.2 ± 29.4
G″ (1 Hz)	Pa	27.9 ± 9.2	10.6 ± 3.5	14.6 ± 4.8	29.1 ± 5.3	25.7 ± 8.5
% elasticity (1 Hz)	-	30.6 ± 4.9	69.3 ± 11.1	65.3 ± 10.5	72.4 ± 11.6	77.6 ± 12.4
HA conc. x % elasticity	mg/mL	551 ± 88	1386 ± 222	1633 ± 263	1811 ± 290	1941 ± 310
Tan delta	-	2.3 ± 0.7	0.4 ± 0.1	0.5 ± 0.2	0.4 ± 0.1	0.3 ± 0.1
Dynamic viscosity (1 Hz)	Pa.	12.6 ± 4.2	22.5 ± 7.4	84.6 ± 27.9	110.8 ± 36.6	103.5 ± 34.1
Cohesivity	mg/drop	21.2 ± 3.6	23.5 ± 4.0	40.2 ± 6.9	52.3 ± 8.9	52.5 ± 8.9
Particle size (d 0.5)	µm	-	481 ± 72	425 ± 64	45 ±7	99 ± 15
Soluble fraction	%	103.5 ± 3.1	39.9 ± 15.2	50.2 ± 19.1	27.3 ± 10.4	16.2 ± 6.2
Swelling factor	%	n.a.	120.6 ± 36.4	400.1 ± 61.0	450.1 ± 96.2	423.3 ± 38.4
Degree of modification	% mol/mol	0	0	4.1 ± 0.1	5.2 ± 1.2	8.5 ± 0.7

**Table 3:** Extrudability parameters of Hyal System dermal products (1.8%, ACP, DUO, LIP and VOL with needle reference.

Parameter	Unit	Hyal System						
		1.8%	ACP		DUO	LIP		VOL
Needle size	-	30 G × 1/2″ (0.3 × 13 mm)	30 G × 3/16″ (0.3 × 4 mm)	30 G × 1/2″ (0.3 × 13 mm)	27 G × 1/2″ (0.4 × 13 mm)	27 G × 1/2″ (0.4 × 13 mm)	27 G × 3/4″ (0.4 × 19 mm)	27 G × 1/2″ (0.4 × 13 mm)
Average extrusion force	N	7.1 ± 0.2	7.9 ± 0.7	10.6 ± 0.5	11.7 ± 0.3	12.9 ± 0.7	17.1 ± 0.1	15.4 ± 0.4
Extrusion force slope	N/s	0.4 ± 0.1	1.1 ± 1.0	1.4 ± 0.6	0.7 ± 0.1	4.6 ± 1.5	5.2 ± 1.1	5.0 ± 4.3

## Data Availability

The data presented in this study are available on request from the corresponding author.

## Supplementary Materials

The following supporting information can be downloaded at https://www.mdpi.com/article/10.3390/gels9090733/s1, Figure S1: Soluble fraction and percentage elasticity of HA hydrogels with HA reaction concentration from 65 to 300 mg/mL (A). Degree of Modification and elasticity of HA hydrogels with starting molecular weights (200, 700 and 1800 KDa) (B).; Figure S2. Representative images (magnification 100X) of murine fibroblasts migration after treatments with HA 200 KDa, HA 700 KDa and crosslinked HA after 6 hours.
