# Supplementary material for: Investigation of Crosslinking Parameters and Characterization of Hyaluronic Acid Dermal Fillers: From Design to Product Performances
Source: Gels. 2023 Sep 9;9(9):733. doi: 10.3390/gels9090733 (PMC10530960; doi:10.3390/gels9090733)
Supplement: Supplementary file 1 [file gels-09-00733-s001.zip › gels-2569774-supplementary.pdf]

Supplementary Materials

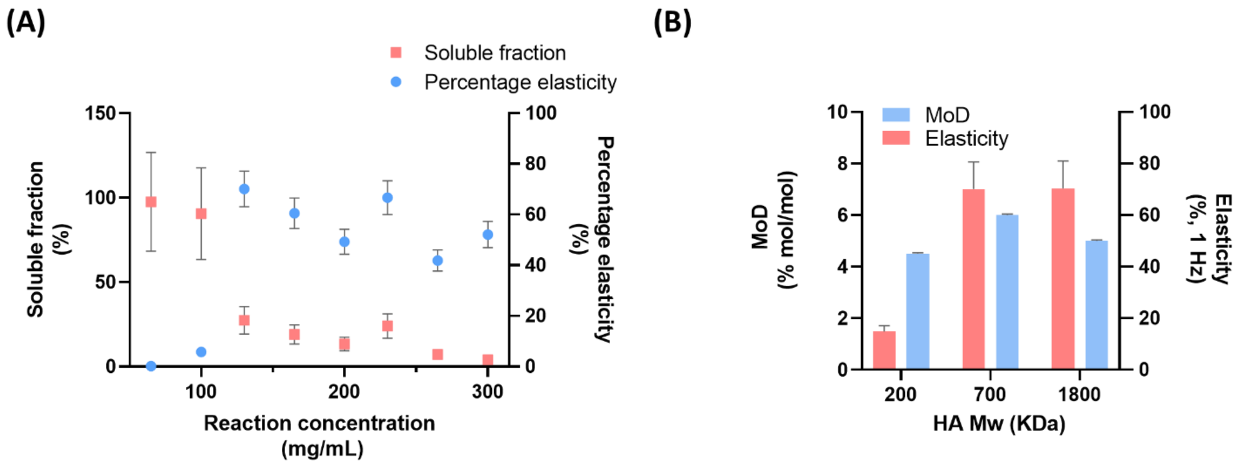

Figure S1. Soluble fraction and percentage elasticity of HA hydrogels with HA reaction concentration from 65 to 300 mg/mL (**A**). Degree of Modification and elasticity of HA hydrogels with starting molecular weights (200, 700 and 1800 KDa) (**B**).

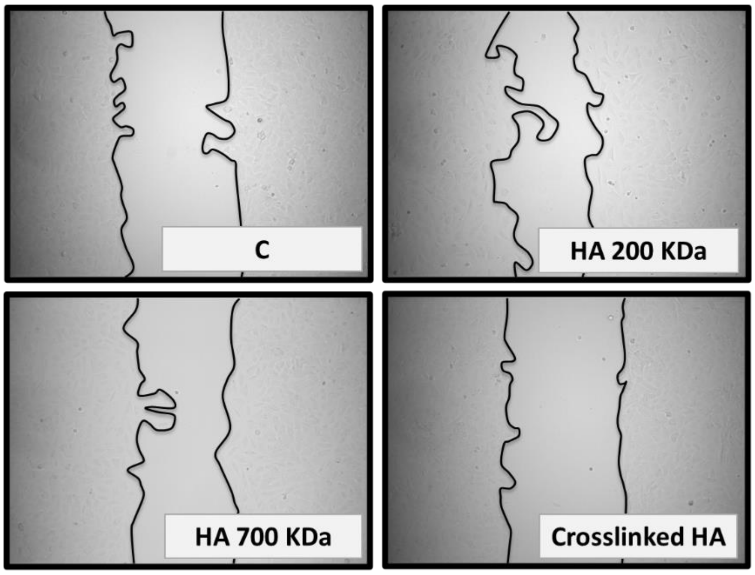

Figure S2. Representative images (magnification 100X) of murine fibroblasts migration after treatments with HA 200 KDa, HA 700 KDa and crosslinked HA after 6 hours
